# The Dublin Hospital Reports and Communications, in Medicine and Surgery

**Published:** 1818-04

**Authors:** 


*The Dublin Hospital Reports and Communications, in Medicine and Surgery, Vol. 1st. Dublin,* 1817.

THE volume is divided into two parts. The first comprehends Hospital Reports; the second, Miscellaneous cases. Should it meet with a favourable reception, it is designed to publish one annually.

ART. 1. *Report of the Hardwicke Fever Hospital for the Year ending the* 31*st of March,* 1817. By JOHN CHEYNE, M. D. Professor of the Practice of Physic in the School of Surgery in Ireland.

The subject of "Fever" has engaged the attention of authors and practitioners from the earliest dawnings of medical science; from the age of Hippocrates to the present: yet it must be confessed, that on many points respecting it, our knowledge is extremely limited, and enveloped in the mists of uncertainty. Theory has succeeded theory, in rapid march; tyrant like, they have slain their "thousands," and sunk into merited oblivion, to be followed by others no less erroneous or destructive. Even in these more enlightened days, when the sun of science seems fast approaching its meridian, the medical world remain divided in their opinions respecting the proximate cause of this affection of the system. One detects it in the brain; another in the medulla spinalis; a third in the abdominal viscera or alimentary canal. Probably, like the travellers, who disputed about the colour of the chamelion, "all are right, and all are wrong." Nothing but patient investigation, in tracing effects to their causes, will extricate us from the labyrinth in which we are bewildered; it is, therefore, the essential duty of every one to contribute his portion of experience to the general stock: Dr. Cheyne has done so, in a manner that evinces the industry and ardour with which he pursues the improvement of his profession.

The Report is prefaced with "an Account of the Hardwicke Fever Hospital;" some "Instructions for the Nurses;" and the "Dietary" of the Institution. The prevailing varieties of fever are described, "without presuming to affix a name to them;" commencing with those which occurred in the latter end of the spring, and summer of 1816.

*Symptoms.* Rigors, flushings, thirst, anorexia. In fifty-three cases out of 80, much head-ache; in three, delirium, which in one instance ended in stupor. A fourth had pain in the bones. An equal number, stitches in the chest, with cough and oppressed breathing, relieved by expectoration, which in one only was bloody. Oppression at the præcordia; nausea, and vomiting. Tongue throughout, grey, with florid edges; urine high coloured; bowels confined. Petechiæ confined to the neglected cases.

*Treatment.* When the eyes were blood-shot, head-ach severe, countenance flushed, pain and tightness in the chest; when there was nausea, vomiting, fullness at the epigastrium; or yellowness of the skin with pain in the right hypochondrium, or irritability of stomach, or diarrhœa with tenderness of belly—the lancet was "a never failing means of relief." It often "strangled the disease in its birth;" and when threatened with relapse, restored the patient to health in a few hours. The appearance of petechiæ, or calor mordax, or want of size in the blood, prevented not its use. Where it was performed at too late a period, or during the exacerbatio critica, it did harm.

Cathartics are next in importance to blood-letting. The use of these in fever has become universal. "By the origin of that practice in Edinburgh, compensation has been made for the fatal errors which were founded on the doctrines of fever so long taught in that city." Mild purgatives were found most efficacious.

Emetics were given, 1st, in cases of threatened relapse, followed by an anodyne and a purgative in the morning; 2d, in the advanced stage, when the tongue was dry and shrivelled, "to resuscitate secretion;" succeeded by cordials.

Opium was administered after the violence of the fever had subsided, the bowels free, the tongue and skin moist.

Blisters in the neighbourhood of the affected organ; especially on the eve of a critical day. Sinapisms in one or two instances where there was affection of the lungs, with typhoid debility.

In the month of August some cases seemed inclined to degenerate into dysentery: In one or two, the fever ended favourably after a discharge of blood from the bowels. The character of the fever changed in September. Added to the flushings, head ache, &c. there were distressing sickness, bitterness of the mouth, yellow and furred tongue, petechiæ, excessive debility. In the treatment, general blood-letting was less efficacious; local bleeding was preferred; emetics of ipecacuan, saline diaphoretics, diluents, and ablution of the head and shoulders.

The fever ranged still more severely in October; with the symptoms above enumerated, great heat of surface, and covered with a florid rash intermixed with papulæ; violet or florid petechiæ; syncope. Severe cases did not terminate before the second week. Recovery, which was tedious, promoted by laxity of bowels, and nocturnal sweats. Relapses frequent. Some sunk unexpectedly, their apparent distress bearing no proportion to the danger. The patient would seem recovering, and take his food regularly; but regained neither flesh nor strength, and wished not to leave his bed. The pulse quick; belly painful and sore; slight fulness at the epigastrium; bowels loose; countenance pale and sunk; mind without energy; involuntary discharge of urine and fæces; hiccup and death. On dissection, the mucous membrane of the alimentary canal had evident marks of previous inflammation. This was the only part uniformly found diseased in this fever. Opiates and cordials accelerate death.

During the last months of 1816, there were many cases of idiopathic dysentery. In the treatment, cordials were sparingly used. Mild purgatives; bleedings of eight or twelve ounces, and blisters to the abdomen. Mercurials were not found so useful as expected; they were therefore laid aside, and flannel bandage, the warm bath, pulv. ipec. comp. castor oil, laudanum, and chalk substituted.

Two cases of the fever were attended with obstinate hiccup; one was treated with stimulants, and died. On dissection, the inner surface of the stomach was found of the colour of mahogany, and coated with a brownish yellow mucus. The other, by leeches, and a blister to the epigastrium, purgatives and refrigerants, and recovered.

Some of the fever patients, especially the more aged, fell into a state of helpless debility, with harsh hot skin, quick pulse, chlorotic complexion. Dissection pointed out inflammation of the mucous membrane of the stomach and intestines. Cordials are here improper: our hopes must rest on leeches, and blisters to the abdomen.

In the last week of October, and first of November, three patients were admitted labouring under Cullen's "typhus gravior." They all died: one on the 11th; another on the 13th; and the third on the 18th day. They had a measly efflorescence on the skin; great debility; stupor; flushed face; dull, inflamed eye; delirium, ending in muttering and coma; subsultus; black and dry tongue; cold extremities; involuntary stools. The effects of vascular activity were evident in the brain, and mucous membrane of the small intestines and bladder.—Bleeding, to be of use, must here be employed early; in more advanced stages, dilution, cordials, blisters; cold to the head, and warmth to the feet, with lax bowels. About the middle of November, convalescence was often interrupted by mucous stools and tormina, which proved fatal to those not blooded in the beginning. Hæmorrhage from the intestines often proved critical.

A patient was admitted on the twenty-second day of fever, with quick pulse, white tongue, pale countenance, emaciation, and cough. On the twenty-sixth and twenty-seventh days he appeared to mend; but on the evening of the twenty-seventh he had a rigor, and next morning was dying of peritoneal inflammation, the contents of the ilium having escaped into the abdominal cavity through an ulcerated aperture.

A diarrhœa stopped suddenly in another; the belly became tumid and tense, the pulse small and quick; the bowels obstinately constipated. Extreme debility and stercoraceous vomiting closed the scene. Coagulable lymph covered the small intestines, and adhered to the peritonæum; the mucous membrane in the state so often described; great intestines studded with red eminences, as in dysentery.

In the first fortnight of January, symptoms of bronchial inflammation were superadded; complexion purple; eye glossy; wheezing and laborious respiration; suffocative fits of coughing; difficult expectoration of viscid mucus; head-ache; weight at the epigastrium; prostration of strength; pulse neither hard nor quick. Petechiæ in one instance. Its favourable termination was in expectoration; its unfavourable, after the manner of peripneumonia notha.

*Dissection.* The lungs did not collapse; mucous membrane of a bright red, and thickened; thus diminishing the diameter of the bronchial canal: secretion from it viscid; in one case it had the colour of pus. Stomach and intestines as before described.

*Treatment.* Small repeated bleedings seemed more appropriate than large; but ipecacuan, calomel, ammonia, seneka, squills, and camphorated tincture of opium were more relied upon.

A case of sudden death occurred during convalescence, in rather a worn out subject, "whose fever had been accompanied with pulmonic inflammation." On the night previously, he disturbed the ward with his talkativeness, and in the morning made his own bed just before his decease. No morbid appearances, on dissection, sufficient to account for death.

In the latter end of January, fevers similar to those of the beginning of this Report, became common, but were oftener attended with petechiæ, brown dry tongue; affections of the sensorium, and early debility. In the first part of February there were three kinds of fever coexistent in the wards.

1st. A few similar to those prevailing all the winter. 2d. Six or eight where there was dejection, prostration of strength, pale or muddy complexion, pungent heat and petechiæ; tongue "as if slightly powdered with chalk," tremulous, and when more advanced, shrivelled, and brown; bowels confined; urine pale; vertigo, delirium, or fatuity. At the end of the second week soporose. In three or four days more, sleep natural, stools loose, urine better. The skin seldom yielded till convalescence had advanced. Œdema of the extremities in three cases; one was bled, another cupped; all recovered.

3d. Such as most physicians would term "synochus." The expression anxious, complexion high, skin hot, petechiæ; tongue white, thirst, confined bowels, scanty high-coloured urine; intellect frequently impaired. Many expectorated bloody mucus. Upon the declination, vesicles appeared about the hands.

In March, the former gained upon the latter kind of fever. Flushing of the face, suffusion of eyes, dun petechiæ, were more general attendants. From those who had cough and determination to the head, ten ounces of blood were taken, with benefit. Bowels kept regular with calomel, head shaved and sponged with cold vinegar and water, feet fomented, and blisters. About the 11th or 12th day, wine.

February, a patient came in with a fever, differing from all that have been described; without flushing, the heat of the skin 108°; the tongue preternaturally clean, and soon aphthous. Pain in the bones; dry cough and difficult expectoration. Convalescence in the middle of the fourth week.—Two similar cases occurred in November.

Some observations follow, on the table annexed to the Report, which shows the number of admissions, deaths, quantity of wine allowed, and number of blood-lettings practised. About half the patients were let blood, leeched, or cupped; in many it was repeated twice or thrice: the averaged quantity of each bleeding ten ounces.

Now, when medical men begin to think for themselves, and throw off that blind attachment to scholastic dogmas which has so long bound their minds with the cords of prejudice; when they no longer fear that fancy-created phantom, "debility,"—the practice of blood-letting in fevers is becoming more general. When judiciously used, it certainly is one of the most valuable, most powerful, most successful remedial measures we possess.

The remainder of the communication is occupied by a detail of many of the cases and dissections which form the basis of the preceding Report: they are by no means the least interesting or valuable part, and we regret our limits oblige us to pass them over.

Art. 2. *On Mania, &c.*

### PART II.

Art. 3. *On the Distortion termed Varus, or Club-Feet.* By A. Colles, M. D. one of the Professors of Anatomy to the R. C. S. Ireland.

As fittest subjects for experiment, Dr. Colles selected children of one or two years of age; and after putting in practice all the most approved plans of treatment recommended in this affection, is led to prefer the following apparatus, which, he says, can be applied so early as a fortnight after birth, and will effect a cure in the space of three months.

It consists—

"—of a shoe made of chamois leather doubled, and having a sole of strong tin interposed. The tin is cut to the size of the sole of the foot, having, however, two small projections left, one opposite the ball of the great toe, another opposite the outer ankle. Each of these has a longitudinal slit, to receive the shouldered end of a splint. The splints are made of tin completely covered with chamois leather, except merely the shouldered end of each, which serves as a kind of tenant to pass down through the mortice-like slit in either projection, and which should be left of such a length as to admit a hole capable of receiving a bit of narrow tape below the sole of the shoe. The splint for the outer side of the limb is a narrow strip of uniform breadth and shape; that for the inner side somewhat resembles the hosier's stocking-leg. They should each be an inch broad, and reach where the leg swells out below the inner condyle of the femur. The upper leather of the shoe is open in front, with holes for the passage of a double lace, which is passed cross-ways from the toes to the instep. The point of the shoe is completely open, so that the toes are entirely exposed. To the heel are sewed two leather straps, long enough to cross once or twice on the instep."

After this description, it is unnecessary to detain our readers with the method of application; the different contrivances about the apparatus will readily suggest their appropriate situations.

A case is related, where there was deformity in both ankles, and displacement of the patellæ, cured by this apparatus in seven months; but the age of the patient is not mentioned.

After the period of infancy, this plan is "much less efficient," and inferior to that of Scarpa; but even with this we shall be often disappointed, especially where the bones themselves are distorted or mis-shapen, which the Doctor affirms, contrary to the opinion of the Italian Professor, is sometimes the case; and in support, relates a case where the deformity in the osseous fabric of the tarsus is very remarkable, and forms the subject of one of the plates attached to this volume.

Dr. Colles concludes with observing, that—

"—the prospect of effecting a cure in varus will be greater in proportion as the subject is younger; and that it can be accomplished with greater ease, and in a very short time, when undertaken immediately after birth."

Scarcely a day passes that we do not, in our ambulations, meet with many subjects of this deformity to excite our commiseration; and on inquiry, we fear, most of them will be found to owe their present unsightly appearance to the want of proper and early applied remedial measures. The necessity of minutely inspecting the limbs of infants immediately after birth, cannot, therefore, be too strongly impressed on the minds of obstetrical practitioners, parents, and nurses.

ART. 4. *Observations on the Remittent Fever and on the Plague which prevailed in the Island of Corfù during* 1815 *and* 1816. By WILLIAM GOODESON, M. D. Assistant Surgeon of the 75th Regiment.

This paper consists of extracts from two letters.

A bilious remittent fever prevailed amongst the troops during the hot weather. It was often attended with yellow skin and irritable stomach, and in the author's opinion differed only in degree from yellow fever. In the early stadium decided advantage was derived from opening the temporal artery, which "cut the disease short at once." The appearances on dissection were — gall-bladder distended with ropy bile; traces of inflammation in the brain; water in the ventricles; vessels of the pia mater turgid; and in three cases, effusion of coagulable lymph under the vertex. There were no marshes near the quarters of the troops. The thermometer stood at 84°. Bad cases were sure to occur after light rains. On the weather getting colder, the fever gradually changed to intermittent. The bark "will cure two-thirds of the cases of intermittents to a certainty;" but in remittents it does harm. Arteriotomy, purgatives, James's powder, effervescing mixture, and opium, with tepid ablutions, were used to combat this last form of fever. Calomel "did mischief, by increasing the action of the liver, and producing tympanites."

Dr. G. had charge of half the district of Lower Leftimo when it was infected by the plague, but saw only twenty cases. — *Symptoms.* Nausea, sallow complexion, rigors, vomiting; tongue loaded with a white curdy matter, its edges red; countenance horribly expressive of despair; staggering, which formed a decided feature of the disease, and was quite sufficient for its diagnosis. Some died delirious, others comatose. Buboes and petechiæ were frequent, large, and numerous. Most died within 48 hours. Mortality, 14 out of 15 All acute diseases were suspended during the prevalence of this, but beyond the limits of the contagion they were rife. The origin of the contagion could not be ascertained; the Doctor thinks it was imported. It owed its dissemination to the ceremony of dis-interring some of its earliest victims, to appease an "angry ghost," which the superstitious inhabitants believed to be the cause of the epidemic.

**Art. 5.** *An Account of the Removal of a Tumor situated beneath the Angle of the Jaw.* By J. W. Cusack, M.D. &c.

A tumour, the size of a hazel nut, appeared beneath the angle of the jaw, and in a few months attained the bulk of an egg. It became lobulated, and went on increasing till it reached from the ear to the lower border of the thyroid cartilage; and from beneath the sterno-mastoideus, to a short distance beyond the middle line of the throat, impeding respiration, articulation, and deglutition.

In the operation two flaps were formed, by first making an incision along the course of the sterno-mastoideus, and a second from the middle of this parallel to the base of the jaw and towards the chin. These flaps were dissected back. Much difficulty was experienced in the attempt to detach the tumour, as the slightest movement of it caused great distress in respiration. The external carotid, which was pushed from its natural position, and lay on the outer edge of the tumour, was now secured by ligature; the digastric and stylo-hyoid muscles, which lay in the way, divided, and the tumour, together with a quarter of an inch of the corner of the os hyoides, to which it adhered, removed by piecemeal. Sutures and simple dressings soon completed the cure.

**Art. 6.** *Account of an Epidemic Petechial Febricula.* By Edward Percival, M.B. Cantab. & Dub. &c.

This epidemic appeared at Delgany, in December, 1814, at a female school. It commenced with anorexia, chilliness, and thirst, unattended with pain in the head or limbs, and so mild as not to prevent the children from following their usual avocations. Each had a copious eruption of dark-coloured petechiæ all over the body on the second day. Duration of the fever nine days, that of the petechiæ five. Its origin could not be traced. Treatment consisted of gentle purges occasionally.

The common doctrine of inflammation, or that of putridity, do not explain the proximate cause of petechiæ:

"Were I to indulge any conjectures on the subject (says Mr. P.) they would be founded on the disturbed balance of the venous and arterious systems; laxity of the subcutaneous capillary veins, and defective absorbent power of the capillary arteries."

**Art. 7.** *Some brief Notices, &c.*

ART. 8. *Observations on Hernia.* By CHARLES H. TODD, M. R. S. I. &c.

It has fallen to the lot of Mr. Todd, within the last five years, to perform the operation of strangulated hernia thirteen times, and to have dissected many cases of this disease. The difference of structure and density of the coverings of the hernial sac does not meet with sufficient attention from junior practitioners, but it is a subject of "paramount importance" to the operator.

In scrotal hernia, immediately under the integuments, is the subcutaneous fascia, varying considerably in thickness, laminæ, and vascularity. The next covering is formed of a fascia derived from the edge of the external abdominal ring united to the cremaster, and adjacent cellular tissue. In old hernia, especially where trusses have been worn, this is frequently so much thickened as to appear composed by strong fibrous bands, and resists reduction. The cremaster muscles are sometimes considerably increased in strength, at others indistinct. The quantity of parts situated between this last named muscle and the hernial sac, of course depends on the relation the hernia bears to the spermatic cord. The following deviations from the usual position of parts is worthy of notice.

In a cadavre with a large scrotal hernia, the spermatic cord, in an undivided state, was extended across the upper part of the sac to its pubal side, and then descended on that side to the posterior surface, where the testicle was situated. Thus the hernia, instead of passing anterior to the cord, passed between it and the inferior pillar of the ring. It is necessary the surgeon should have in mind the possibility of this occurrence, and be careful to *feel* his way as he proceeds in the operation.

Mr. Hey's opinion respecting the formation of the "hernia infantilis" is erroneous. "In no instance is the sac in actual contact with the testicle." It protrudes "completely within the cellular sheath of the spermatic cord, and it descends near to the point of insertion of these vessels into the testicle;" its fundus "comes in contact with the upper part of the tunica vaginalis testis, and receives from it, on its lower surface, a serous covering, proportioned to the magnitude or degree of distention of the sac." This species is not peculiar to infancy; a case is related which occurred in an adult.

To distinguish crural hernia from inguinal is sometimes difficult, but the diagnosis is of the last importance. The neck of the tumour should be searched for under the crural arch, with the thigh bent. In the anatomy of this hernia the fascia propria claims our attention. Mr. Todd conceives it to be the "continuation of that fascia which, in Mr. Abernethy's operation for iliac aneurism, prevented his passing his fingers beneath the artery."

The peculiarity of the following case deserves record. —An elderly man was admitted into Richmond Surgical Hospital on the tenth day of strangulated hernia. "A flattened tumour was situated on the right side of the abdomen, extending from the bend of the thigh almost to the umbilicus, and from the pubis to the anterior superior spinous process of the ileum. The skin exhibited an erysipelatous redness, with a desquamation of the cuticle; in the centre of the tumour a fluctuation was felt, and a crepitus towards its circumference." Pulse scarcely perceptible, extremities cold. The hiccough and vomiting ceased the preceding evening. Two small evacuations per anum. Abdomen tense, and painful. An incision was made into that part where the fluctuation was evident, about three inches above the crural arch, and nearly half a pint of fœtid matter discharged. The finger was now passed in, and the wound enlarged downwards, when a dark coloured hernia, the size of a walnut, was discovered at the femoral ring. All the surrounding cellular substance had mortified. After the sac was opened, the intestine (sphacelated) adhered to its neck, and a small opening was made in it. At the expiration of a month he was discharged well, except a small fistula at the groin, through which a quantity of bilious fluid occasionally passed.

With respect to the seat of stricture in femoral hernia, Mr. Todd is decidedly of opinion that it is caused by the "sharp aponeurotic edge which forms the boundary of the femoral ring;" and that it is more safely and easily removed by a cautious division towards the pubis than in any other direction.

Art. 9. A Case of Melæna, &c.

Art. 10. *Of Jaundice, unaccompanied with any discoverable Disease of the Liver, or Turgescence or Obstruction of the Biliary Ducts.* By J. Cheyne, M. D. &c.

An American, aged 17, was admitted on the ninth day of fever. Tunica adnata and skin of a deep yellow; low delirium; vomiting of dark fluid; involuntary discharge of urine, and dark fœtid fæces. He died five days after.

*Dissection.* Alimentary canal and biliary system healthy; gall bladder contained light coloured bile; kidnies enlarged, their exterior "mottled," scarcely any of their original structure remained; coats of the bladder thickened and diseased; effusion of yellow serum between the membranes of the brain; ventricles containing several ounces of yellow fluid, their inner surface covered with spots resembling petechiæ.

Dr. C. considers this as a case of typhus attended with jaundice. The treatment consisted in the exhibition of mild mercurials and wine: but, should a similar case occur, the wine would be omitted, and blisters to the head and nucha, pediluvium, Dover's powders, with calomel, made use of.

After a protest against the empirical use of mercury in hepatic affections, and citing two instances in which this mineral actually produced jaundice, we have the following case.

Sarah Armstrong, ætat. 26, had numerous subcutaneous tumours on different parts of the body; nocturnal pains; enlarged joints; emaciation. Nine months before this, she had chancres which got well by a few mercurial pills. She was for six months treated as pseudo-syphilitic, and every symptom disappeared. The complaint returning, smart ptyalism was established; oppression, nausea, jaundice, bileless fæces, followed. The mouth was nearly well; she was suddenly seized with a kind of epileptic paroxysm with vomiting of a bloody fluid, and died two days after.

*Dissection.* A yellow tinge pervaded the solids and fluids of the body. Vessels of the brain turgid; effusion between the membranes. An hydatid near the pineal gland; ecchymosis at the base of the brain. The liver small; its concave surface pale, red, and mottled; its convex surface, uniform dark purple, and connected to the duodenum by extensive membraneous adhesions of long standing. Gall bladder flaccid, containing mucus tinged with bile. Coats of the stomach thickened; inner membrane covered by a tenacious mucous; its cavity contained a coffee-coloured fluid.

Art. 11. *On the Cause of the Disease termed Trismus Nascentium.* By A. Colles, M. D. &c. &c.

Various have been the conjectures offered as to the cause of this affection.—The following are the appearances which presented themselves on dissection.

"On cutting into the abdomen, the peritonæum covering the umbilical vein was highly vascular, as if from inflammation; this extended sometimes up to the fissure of the liver; often, however, not for a greater length than one inch above the umbilicus. The peritonæum, in the course of the umbilical arteries, appeared still more influenced; an appearance which extended often as far as the sides of the bladder. Besides the appearance of the peritonæum along their posterior surface, the cellular substance which covered them, and the urachus anteriorly, was loaded with a yellow watery fluid, even down to the bladder. Leaving the umbilicus untouched, if we cut open the umbilical vein, from the liver to the vicinity of the umbilicus, we found only a few small coagula of blood within the canal; the inner surface of the vein was pale, and free from any marks of inflammation, yet the coats of the vein altogether were much thickened. The umbilical arteries exhibited evident marks of inflammation. 1st. On slitting them up, a thick yellow fluid, resembling coagulable lymph, was found with their coats. 2d. In all instances their coats were much thickened and hardened, even as far as the fundus of the bladder. On cutting into the umbilicus itself from its posterior or peritoneal surface, we found in the centre a space, about half an inch long, occupied by a soft yellow substance, which bore a very strong resemblance to coagulable lymph, produced by inflammation; it was this which formed the prominence observed in the external veins of the fossa."

Dr. C. has been informed by a lady who resided many years in Jamaica, that the means of prevention adopted by the Negroes of that island, among whom the complaint was formerly very prevalent and fatal, were, to plunge the infant into a cold bath for nine days, and to dress the cord with spirits of turpentine.

ART. 12.—*Case of Dropsy by Conversion of Disease from the Skin to the serous and cellular Membrane.* By EDWARD PERCIVAL, M. B. &c.

A female of 25, convalescent from fever, relapsed, with unusual debility; pulse frequent and feeble; skin covered with papillæ: previous to admission she had scabies purulenta. She recovered her strength a little, and then the belly and limbs suddenly swelled surprisingly; the respiration became oppressed; the florid hue of the papillæ nearly disappeared; the scabious pustules dry and shrunk; bowels constipated. By digitalis, &c. the limbs returned to their natural size, but the patient sunk.

*Dissection.* Fluid in the thorax and pericardium. Pleura inflamed. Right lung unusually dense. Heart larger and softer than usual. Intestines distended with air, and studded with livid spots. Serous membranes of the abdomen and viscera inflamed. Signs of congestion in the liver, pancreas, and spleen. Vessels of the brain turgid; pia mater florid. Slight effusion in the ventricles.

ART. 13.—*History of a Wound of the Neck, in which the Operation of tying the common Carotid Artery was performed with Success.* By JOHN BROWNE, M. D. &c.

THE common carotid was injured in a punctured wound inflicted with a pen-knife. It was secured with a double thread ligature below the orifice only, and the patient did well.

ART. 14.—*A Case of Ruptured Intestine.* By CHARLES H. TODD, Member of the Royal College of Surgeons, &c.

THE subject of this case was two years old. Symptoms of ileus appeared immediately after falling from a chair. The extremity of the jejunum was completely torn off from the duodenum, and separated from it nearly an inch. A quart of broth was found loose in the cavity of the peritonæum.

ART. 15.—*On the Virtues of James's Powders in the Apoplectic Diathesis.* By J. CHEYNE, M. D. &c.

No doubt this medicine may be of use in these affections; but, in the words of Dr. Cheyne, it must not "supersede the usual remedies; namely, bleeding, purgatives, and a strict antiphlogistic regimen. They are merely a part of the prophylactic treatment, to be employed when the fit or threatening of apoplexy is over."

ART. 16.—*History of an Enlargement of the Biliary Duct.* By C. H. TODD.

A girl, ætat. 14. Skin orange colour; emaciation; anasarca; pain; sensibility to external objects; hands firmly clenched; jaws fixed; incessant moaning and frequent, screaming. Abdomen distended with fluid, particularly the hypochondriac regions. A little below the ensiform cartilage, to the right of the linea alba, a fluctuation was evident—supposed to be that of an abscess of the liver. From an opening made with a lancet, a thin fluid, coloured with green bile, discharged. Two quarts more were drawn off through a canula: some little relief seemed to follow, but she died in the evening, comatose.

*Dissection.* The hepatic and common ducts were enormously distended, containing more than a quart of the same kind of fluid already mentioned. The ducts formed a sac extending from the porta of the liver to the sacrum, lying behind the duodenum and pancreas, and covering the anterior surface of the right and left kidney. The pancreas was scirrhous, and converted into a hard, solid mass.

ART. 17.—*On Periostitis, or Inflammation of the Periosteum.* By P. CRAMPTON, M. D. F. R. S.

The practical conclusions to be drawn from the cases related in this paper, are thus summed up by the author:

1. Periostitis, unconnected with specific disease, is frequent, and to be distinguished from the venereal by accurate investigation only.

2. In the *acute* form, anti-inflammatory measures are necessary, and division of the inflamed membrane down to the bone.

3. In the *chronic* the treatment is similar.

4. In the constitutional treatment, attention to the digestive organs, sea bathing, country air, and sarsaparilla are proper.

From the extended analysis which we have given of this volume, the reader will have already appreciated its value. In one word, a more important or interesting publication has not appeared of late years. It bids fair to surpass the Medico-Chirurgical Transactions.

---

